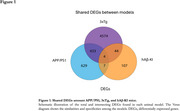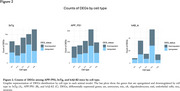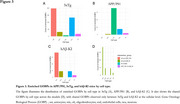# Exploring cell type‐specific hippocampal transcriptional signatures in amyloidosis mouse models

**DOI:** 10.1002/alz70855_105268

**Published:** 2025-12-24

**Authors:** Rodrigo Sebben Paes, Gabriela Mantovani Baldasso, Christian Limberger, Marco Antônio De Bastiani, Eduardo R. Zimmer

**Affiliations:** ^1^ Universidade Federal do Rio Grande do Sul, Porto Alegre, Rio Grande do Sul, Brazil; ^2^ McGill Centre for Studies in Agin, Montreal, QC, Canada; ^3^ Brain Institute of Rio Grande do Sul (InsCer), PUCRS, Porto Alegre, Rio Grande do Sul, Brazil

## Abstract

**Background:**

The growing recognition that late‐onset AD (LOAD) and familial AD (fAD) follow distinct pathophysiological pathways highlights the possibility that different Alzheimer's disease (AD) subtypes could reflect varying molecular and cellular features. However, the precise role of each brain cell type on each AD subtype remains poorly understood. In this context, genetically modified animal models provide a valuable tool to explore AD genetic variability. In this study, we aimed to investigate the transcriptional signatures of cell types in the hippocampus across two fAD mouse models (APP/PS1 and 3xTg) and one LOAD mouse model (hAß‐KI).

**Method:**

We analyzed bulk hippocampal transcriptomics data from the Gene Expression Omnibus repository and the AMP‐AD Knowledge Portal. Gene expression deconvolution was performed using Population‐Specific Expression Analysis (PSEA) to identify differentially expressed genes (DEGs) specific to neurons, astrocytes, microglia, oligodendrocytes, and endothelial cells (FDR‐adjusted *p*‐value < 0.05). Functional enrichment analysis (FEA) of Gene Ontology Biological Processes (GOBPs) was performed for the DEGs from each cell type using the enrichGO function in the clusterProfiler R package (v3.16.1).

**Result:**

We identified 5,055, 1,073, and 162 DEGs in APP/PS1, 3xTg, and hAß‐KI mice, respectively, with the greatest overlap observed between the fAD models (APP/PS1 and 3xTg models) (Figure 1). Additionally, fAD models revealed higher counts of DEGs in neurons, oligodendrocytes, and microglia. Meanwhile, in hAß‐KI, oligodendrocytes had the highest counts. (Figure 2). At the biological level, neurons consistently demonstrated greater enrichment of GOBPs across all three models. In contrast, microglia showed more enriched GOBPs in APP/PS1, while astrocytes demonstrated more enriched GOBPs in the 3xTg and hAß‐KI models, with 37 shared GOBPs between them (Figure 3).

**Conclusion:**

We found that fAD models had greater transcriptional alterations than the LOAD model, possibly due to the aggressive pathology caused by fAD mutations. Additionally, neurons were consistently affected across all models, highlighting their vulnerability to AD. Overall, the three models exhibited distinct transcriptional and biological signatures, suggesting each one of them is suited to address specific aspects of the AD landscape.